# Diverse pathways of escape from all well-characterized VRC01-class broadly neutralizing HIV-1 antibodies

**DOI:** 10.1371/journal.ppat.1007238

**Published:** 2018-08-20

**Authors:** Yuka Otsuka, Kimberly Schmitt, Brian D. Quinlan, Matthew R. Gardner, Barnett Alfant, Adrian Reich, Michael Farzan, Hyeryun Choe

**Affiliations:** 1 Department of Immunology and Microbiology, The Scripps Research Institute, Jupiter, Florida, United States of America; 2 Informatics Core, The Scripps Research Institute, Jupiter, Florida, United States of America; University of Zurich, SWITZERLAND

## Abstract

Many broadly neutralizing antibodies (bNAbs) against human immunodeficiency virus type 1 (HIV-1) were shown effective in animal models, and are currently evaluated in clinical trials. However, use of these antibodies in humans is hampered by the rapid emergence of resistant viruses. Here we show that soft-randomization can be used to accelerate the parallel identification of viral escape pathways. As a proof of principle, we soft-randomized the epitope regions of VRC01-class bNAbs in replication-competent HIV-1 and selected for resistant variants. After only a few passages, a surprisingly diverse population of antibody-resistant viruses emerged, bearing both novel and previously described escape mutations. We observed that the escape variants resistant to some VRC01-class bNAbs are resistant to most other bNAbs in the same class, and that a subset of variants was completely resistant to every well characterized VRC01-class bNAB, including VRC01, NIH45-46, 3BNC117, VRC07, N6, VRC-CH31, and VRC-PG04. Thus, our data demonstrate that soft randomization is a suitable approach for accelerated detection of viral escape, and highlight the challenges inherent in administering or attempting to elicit VRC01-class antibodies.

## Author summary

Several potent antibodies against human immunodeficiency virus type 1 (HIV-1) have been evaluated in clinical trials. Use of these antibodies in humans, however, is problematic, because easy viral escape remains a major concern. To gain greater insights, we sought to develop an approach to rapidly assess the likelihood of viral escape from such antibodies. We show here that soft-randomization mutagenesis is a suitable approach to introduce a controlled number of changes into defined target regions. As a proof of concept, we used this approach to detect the HIV-1 variants fully resistant to VRC01-class of antibodies. We observed that within a few passages of the soft-randomized library of viruses in the presence of potent HIV-1 antibodies, a remarkably wide array of variants emerged, including variants resistant to every VRC01-class antibody. This study provides insights into a wide range of escape pathways, and describes a method for rapidly assessing the likelihood of viral escape from antibodies or small molecules targeting the HIV-1 envelope glycoprotein.

## Introduction

A large number of potent broadly neutralizing antibodies (bNAbs) have been generated from HIV-1-infected individuals (reviewed in [1–5]). Many of these bNAbs, including 2F5, 4E10, PGT121, VRC01, 3BNC117 and 10–1074, have been or will be evaluated in clinical trials [6–12]. A number of animal studies have shown that administration of bNAbs can prevent infection, and reduce viral loads in an established infection [13–22]. Previous human studies also show that bNAbs can reduce viral loads or delay viral rebound upon treatment interruption [6–8, 11, 12, 23]. However, HIV-1 generally escapes these bNAbs when they are administered to infected animals or humans [6–8, 11–15, 17, 18, 20, 21, 23–26].

Escape pathways of HIV-1 from inhibitors against protease and reverse transcriptase have been comprehensively documented through large-scale studies of patients treated with these inhibitors. In contrast, escape from neutralizing antibodies has not been similarly described, in part because no antibody has yet been tested in a sufficiently large cohort of HIV-1 positive individuals. Most reported *in vivo* escape variants emerged from a limited number of animals or volunteers who participated in clinical trials [6–8, 11, 13, 20, 24–27]. In addition, although most bNAbs have been characterized with large panels of HIV-1 isolates [28–30], these studies do not provide direct insight into the likelihood of viral escape, because most HIV-1 isolates have never been exposed to or selected by these exceptional and rare antibodies. In some cases, the number of avenues to escape may be small because of structural and functional constraints on conserved epitopes of the envelope glycoproteins (Env). Nevertheless, to use bNAbs in humans or to design vaccines based on their epitopes, it is essential to understand how viruses can escape them. Although such information is now available for some antibodies from clinical trials, and other mutations have been observed in animal models or selected in cell culture, only a limited number of escape variants have been described for any given antibody or combination of antibodies. This small number is a consequence of inefficient process by which resistant variants emerge. This process relies on errors naturally generated by the viral reverse transcriptase, which introduces approximately one mutation per newly integrated provirus. Because these mutations are distributed throughout the viral genome, fewer than 1 in 10 of such mutations affect the *env* gene, the target of all bNAbs. Typically, the emergence of resistant variants is much faster *in vivo* than *in vitro*—escape variants usually emerge within a few weeks after antibody infusion [6–8, 11, 13, 23–26]—likely owing to much higher number and diversity of viruses found in each individual compared to that used in *in vitro* experiments. Nonetheless, only a limited number of escape pathways were detected *in vivo*, possibly because once a resistant virus emerges, it soon dominates the viral swarm, eliminating the selection pressure that would otherwise lead to additional resistant mutations. Therefore, to accumulate a meaningful number of escape mutations, this process needs to be repeated great many times or in many individuals or animals.

Several mutagenesis methods have also been adopted to accelerate viral evolution. Of these, the mutation rate of error-prone PCR can be difficult to control, and alanine-scanning mutagenesis is limited to mutating residues only to alanine, precluding other substitutions or combinations of substitutions available to the virus. A library constructed by codon mutagenesis, Exceedingly Meticulous and Parallel Investigation of Randomized Individual Codons (EMPIRIC) and Single-site saturation mutagenesis (SSM) were used to accelerate the evolution of HIV-1 or influenza A virus [31–33]. These methods are comprehensive, but labor intensive and only one mutation is introduced per copy of genome. On the other hand, random mutagenesis would generate high number of mutations, but in an overwhelming number of cases, the resulting Envs would not fold or function properly. Another mutagenesis technique, soft randomization, originally developed for phage display applications, allows generation of libraries whose members have a small, desired number of mutations distributed throughout a target region [34, 35]. This technique permits it by using primers synthesized with nucleotides mixed at a non-equimolar ratio, favoring original nucleotides [36]. The optimal composition and use of a hand mix ratio depend on the number of target codons to be altered, the library size, and how wobble codons are addressed.

Here, we developed a method that can help anticipate a range of viral escape pathways from antibodies with a well-defined epitope. To assess its usefulness, as a proof of concept, we tested this method against the CD4-binding site (CD4bs) bNAbs. We generated a replication-competent HIV-1 library expressing Envs diversified by soft randomization in the CD4bs, and selected the escape variants that were resistant to neutralization by VRC01-class CD4bs antibodies. Soft randomization introduced a controlled number of mutations into the majority of library members, and a large number of antibody-resistant isolates could be identified after only a few passages. Most randomly chosen clones of escape variants were resistant to all well-characterized VRC01-class antibodies, including N6, an exceptionally broad antibody of this class [37], in addition to the antibodies used in the selection. Because the epitope regions of CD4bs antibodies overlap with the CD4 binding sites, the resistant variants exhibit growth kinetics slower than that of the parental virus, as previously shown, but nonetheless they readily outgrew the parental virus at antibody concentrations commonly targeted in clinical trial [8, 10, 38]. Collectively, our data demonstrate that soft randomization can be usefully applied to identifying viral escape pathways in the face of a specific selection pressure.

## Results

### Construction of a HIV-1 proviral library with a soft-randomized *Env*

**Fig 1:**
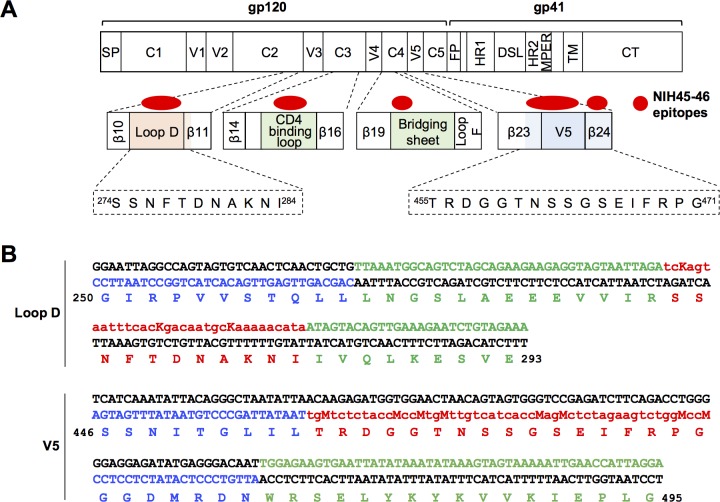
Construction of a HIV-1 proviral library with a soft-randomized *Env*. (**A**) A representation of the HIV-1 *env* gene. Regions containing CD4 binding sites (colored boxes)—Loop D, CD4 binding loop, bridging sheet and V5—are expanded with NIH45-46 epitopes indicated by filled red ovals. Residues of Loop D and V5 regions of the HIV-1 isolate ADA, soft randomized in this study, are shown below. (**B**) Sequences of primers used to soft-randomize the Loop D and V5 regions of ADA Env are shown. Amino-acid numbers are based on HXB2 numbering. Soft randomizing primers were synthesized by hand mixing 88% of the original nucleotide and 4% each of the other three nucleotides (88:4:4:4) for the first two codon positions (nucleotides in lower case) of Loop D residues, and 91:3:3:3 ratio for the first two codon positions of V5 residues. For wobble positions of Loop D residues, an equimolar mix (50:50) of G and T (indicated as K) was used for 4- and 6-codon amino acids. For V5 primer, because it is anti-sense, C and A (indicated as M) were used. Nucleotides subject to soft randomization are colored in red. Both the 5’ and 3′ of the primers outside the randomized regions were extended (shown in green) to have a melting temperature matching the pairing primers (shown in blue).

To determine if soft randomization could generate a useful library of HIV-1 proviruses, we soft randomized the epitope regions of VRC01-class antibodies in the *env* gene, and selected the resulting virus library with the VRC01-class bNAb NIH45-46. We chose NIH45-46 because it was isolated from the same patient from which VRC01 was derived, but it is more potent than VRC01 [39], and because it has been evaluated in a clinical trial. As shown in Fig 1A, the binding epitopes of NIH45-46 are primarily located in the four regions of Env (Los Alamos National Laboratory (LANL) database CATNAP): Loop D, CD4 binding loop, bridging sheet and variable region 5 (V5). To minimize interference with CD4 binding, only the residues in Loop D, V5 and in their vicinity were randomized, whereas the CD4-binding loop and the bridging sheet were left unmodified. The compositions of the PCR primers used to build our library are shown in Fig 1B, and based on the sequence of ADA, a well-characterized clade B R5 isolate. Soft-randomizing primers were synthesized by handmixing original nucleotide and the other three nucleotides at a selected ratio for the first two positions of each codon. To incorporate a small number (1 to 3) of mutations per region, we use a handmixing ratio of 88:4:4:4 for the 11 amino acids of Loop D, and 91:3:3:3 for the longer V5 region (17 residues). An equimolar mix of G and T for the wobble positions of all amino acids encoded by 4 or 6 codons in Loop D, and an equimolar mix of C and A was used for V5 regions, because the soft-randomizing primer for V5 is anti-sense. As shown in S1 Fig, Loop D and V5 libraries were separately generated by PCR amplification of the entire pBR322 plasmid containing ADA *env* gene, pBR322-*Env*(ADA), as a template, and ligated using 5’ phosphate in the soft- randomizing primers. V5 library was subcloned into the plasmid containing Loop D library using an enzyme site engineered between Loop D and V5 regions. The resulting *env* library containing Loop D and V5 regions was then subcloned into the pNL4-3 proviral plasmid carrying ADA *env* gene, pNL-ADA, yielding a proviral library encoding diverse Env proteins, pNL-ADA-Lib.

### Soft randomization introduces a controlled number of amino-acid substitutions into a specific target region

**Fig 2:**
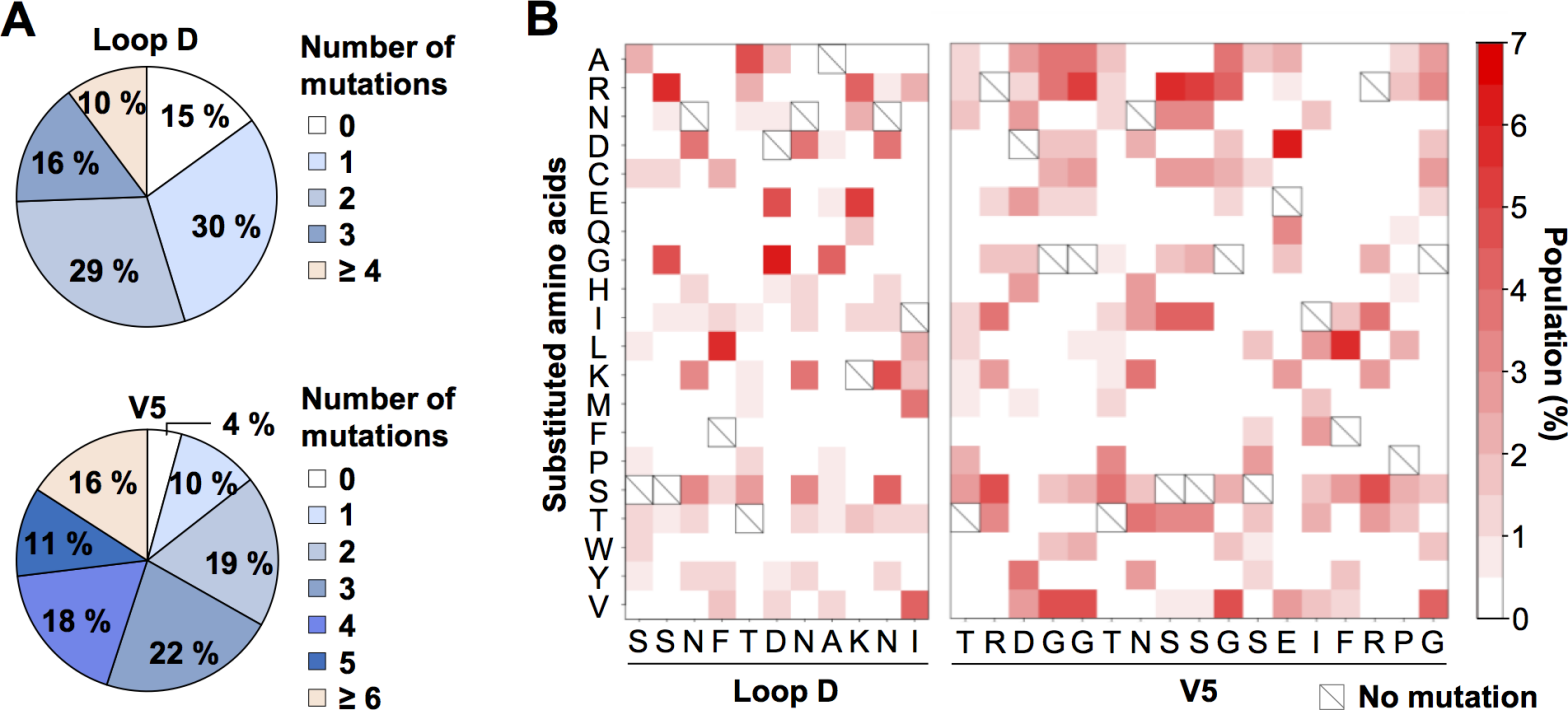
Soft randomization introduces a controlled number of amino-acid substitutions into a specific target region. (**A**) The distributions of the number of amino-acid substitutions in the Loop D and V5 regions are shown. Pie charts are generated based on the data from approximately 600,000 next generation sequence reads. (**B**) Amino acid substitutions in Loop D and V5 regions of the parental virus library are shown in 2-dimensional heat maps. The relative frequency of the substitutions of each residue is represented by a red gradient. The original amino acids of wild-type ADA Env are indicated as a square with a diagonal line.

An NL-ADA-Lib virus population (described here as a “swarm”) was then produced by transfecting HEK-293T cells with the pNL-ADA-Lib plasmid. To assess the composition of the library, a fragment encompassing both Loop D and V5 was amplified by RT-PCR and sequenced by paired-end Illumina MiSeq, and the total number of mutations in Loop D and V5 regions of each library member was analyzed. Only 21% of the total reads contained either stop codons or unknown amino acids, fewer than what is typically observed with less controlled mutagenesis approaches. These reads were excluded before analysis. The most common number of amino-acid substitutions in Loop D and V5 were 1 to 2 and 2 to 4, respectively, typically yielding 3 to 6 substitutions combined (Fig 2A), consistent with our predicted values. The proportion of non-mutated members was relatively low: 15% for Loop D and 4% for V5. More than 74% of the library members contain 1–3 mutations in Loop D, and 69% contain 1–4 mutations in V5, indicating that the majority of NL-ADA-Lib members contain any number of substitutions between 2 and 7. The substitution profile of each soft-randomized residue is shown in Fig 2B. Substitutions of each residue were relatively evenly distributed among 19 amino acids, with substitution biases reflecting one or two shared nucleotides between mutated and parental codons. Thus, soft-randomization can introduce a high degree of controlled diversity in selected regions of a viral genome.

### Only a few passages of library viruses in the presence of a CD4bs bNAb is sufficient for escape viruses to emerge

**Fig 3:**
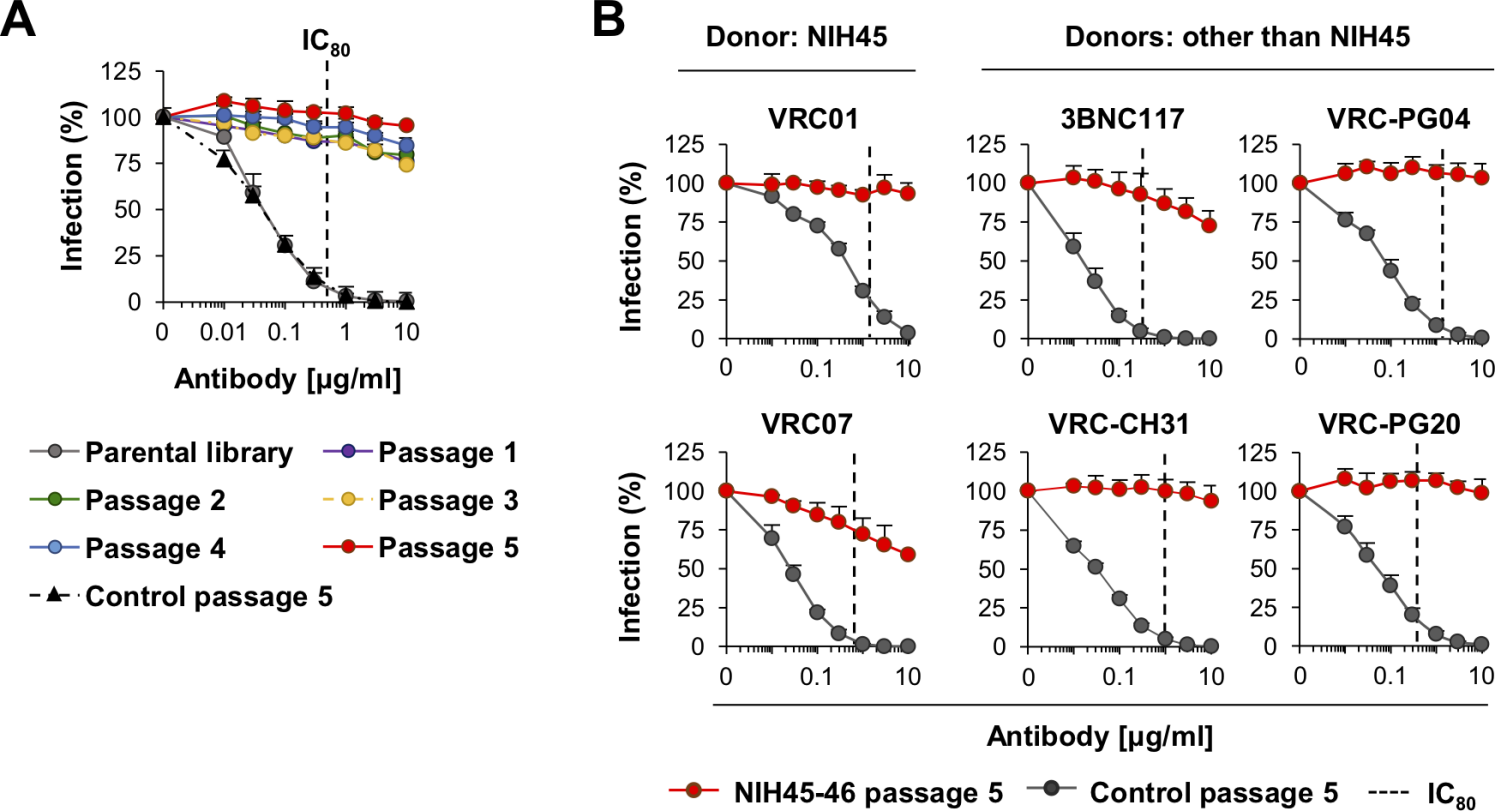
Only a few passages of library viruses in the presence of a CD4bs bNAb is sufficient for escape viruses to emerge. (**A**) Neutralization assays of the passage 0–5 swarms selected against NIH45-46. Assays were performed in TZM-bl reporter cells. ‘Parental library’ is the virus library without any passage, and ‘Control passage 5’ is the parental virus library passaged 5 times in the absence of NIH45-46. (**B**) Neutralization assays with the NIH45-46 passage 5 swarm against the indicated CD4bs bNAbs. VRC01 and VRC07 were isolated from the same donor as NIH45-46 (donor: NIH45), but 3BNC117, VRC-PG04, VRC-CH31 and VRC-PG20 were isolated from Patient 3, Donor 74, CH0219 and IAVI 23, respectively. Averages ± SD of three independent experiments performed in duplicates are shown. Statistical significance was calculated by two-way ANOVA and *p* value was <0.001 for all differences with the relevant control swarm.

To validate that escape variants can be easily detected from the soft-randomized library of viruses, we passaged the library swarm in the presence of indicated concentration of NIH45-46 (S2A Fig). Virus-containing culture supernatants were harvested two days later (NIH45-46 passage 1 swarm), and this process was repeated four additional times at the indicated antibody concentrations (S2A Fig). The virus swarm from each passage was then assessed for their resistance against NIH45-46 in TZM-bl luciferase reporter cells (Fig 3A). Both the parental NL-ADA-Lib swarm and that passaged 5 times in GHOST cells in the absence of any antibody (control passage 5) were used as controls. We observed that a single passage already conferred high level of resistance, and five passages, taking only two days per passage, were sufficient to select near complete resistance. We next assessed the NIH45-46 passage 5 swarm for their resistance to other VRC01-class CD4bs antibodies (Fig 3B). VRC01 and VRC07 were isolated from the same individual from whom NIH45-46 was derived, but 3BNC117, VRC-CH31, VRC-PGV04, and VRC-PG20 were isolated from different donors. Average IC_50_ and IC_80_ values of these antibodies for various HIV-1 isolates are shown in S2B Fig. As shown in Fig 3B, NIH45-46 passage 5 swarm was resistant to all VRC01-class CD4bs antibodies assessed, with the exception of 3BNC117 and VRC07 to a limited degree.

**Fig 4:**
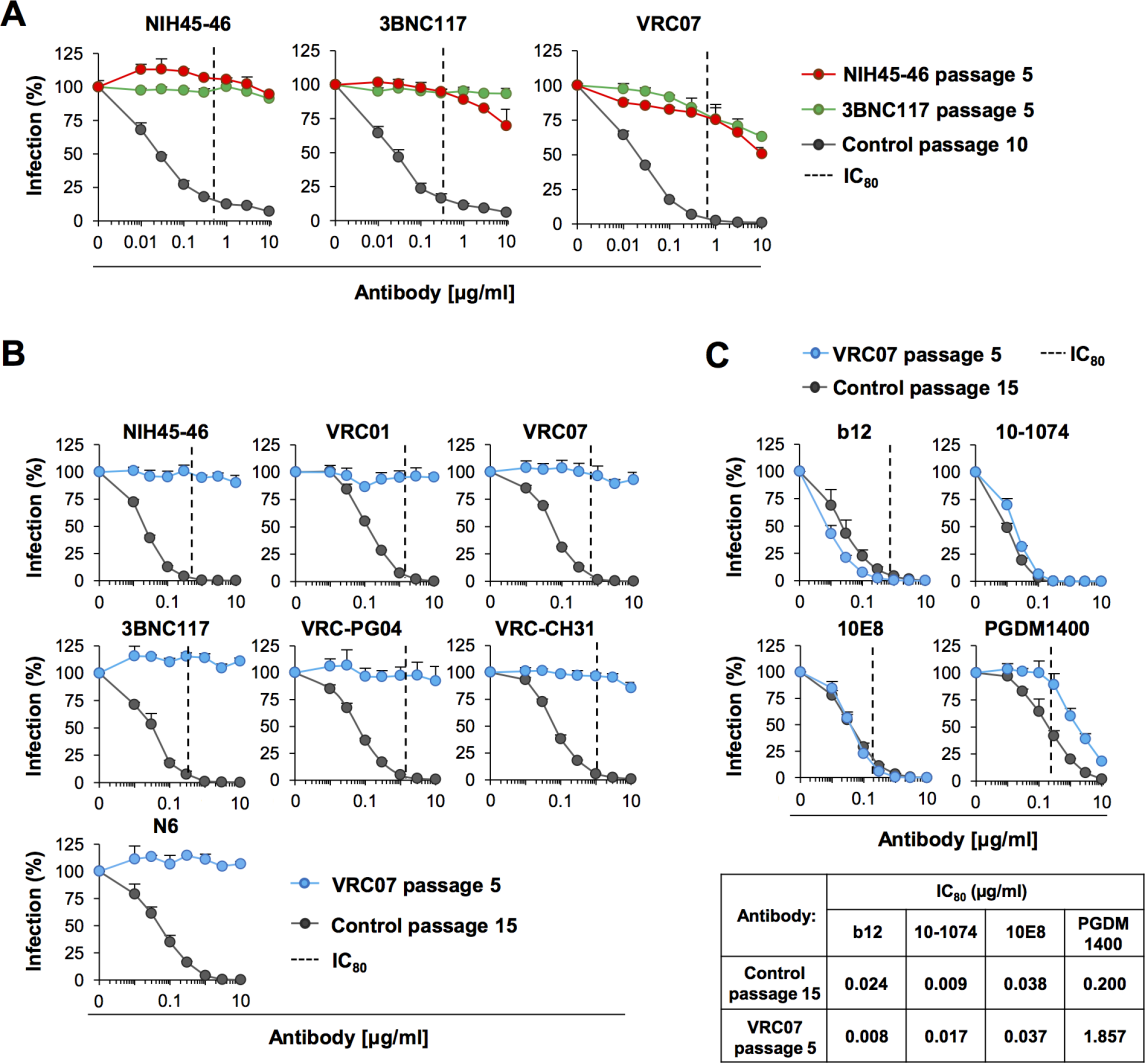
Resistance to one set of VRC01-class antibodies renders the viruses resistant to other antibodies in the same class. (**A**) NIH45-46 passage 5 swarm was further passaged 5 times in the presence of NIH45-46 and 3BNC117 at the concentrations shown in S2A Fig. The resulting 3BNC117 passage 5 swarm was assessed for its sensitivity against the indicated antibodies. ‘Control passage 10’ is the parental library swarm passaged 10 times in total in the absence of any antibody. (**B**) Neutralization assays of the VRC07 passage 5 swarm using the indicated VRC01-class bNAbs. VRC07 passage 5 swarm was obtained by passaging the NIH45-46 passage 5 swarm 5 times each against 3BNC117 and VRC07. As a control (‘Control passage 15’), the parental virus library was passaged the same number of times (15) in the absence of any antibody. Statistical significance was calculated by two-way ANOVA and *p* value was <0.001 for each antibody. (**C**) Neutralizing assays of the same swarm as shown in (B) using non-VRC01 class CD4bs antibody b12 and non-CD4bs bNAbs 10–1074, 10E8 and PGDM1400. 10–1074 recognizes a V3-loop proximal glycan, 10E8 binds the gp41 MPER region, and PGDM1400 recognizes the apex of the Env oligomer. The table below the graphs shows the IC_50_ values of the indicated antibodies, calculated from the neutralization assays, for the control passage 15 and VRC07 passage 5 swarms. Dotted lines in (A-C) represent average IC_80_ values of the antibodies for ADA isolate, and were obtained from the LANL database CATNAP (http://hiv.lanl.gov/catnap). In the case of the N6 and 10–1074, only IC_50_ values are available: 0.074 μg/ml and 0.003 μg/ml, respectively. Averages ± SD of three independent experiments performed in duplicates are shown.

To determine whether complete resistance against all VRC01-class CD4bs antibodies can be obtained, we grew NIH45-46 passage 5 swarm in the presence of 3BNC117. The resulting swarm was then passaged four additional times at the concentrations indicated in S2A Fig. To prevent de-selection of NIH45-46 resistant viruses, NIH45-46 was included in all passages. 3BNC117 passage 5 swarm was then assessed for its resistance to 3BNC117 and VRC07 (Fig 4A). Interestingly, although the virus has become completely resistant to 3BNC117 even at 30 times of its IC_80_, its sensitivity to VRC07 was not changed. We therefore passaged this swarm in the presence of VRC07. The resulting VRC07 passage 5 swarm was resistant to all the VRC01-class antibodies we tested (Fig 4B), including the antibody, N6, a recently isolated CD4bs bNAb with the highest breadth thus far described in the class. We further assessed the same swarm against non-VRC01-class CD4bs antibody, b12, and non-CD4bs antibodies 10–1074, 10E8 and PGDM1400 (Fig 4C). VRC07 passage 5 swarm was sensitive to all of these antibodies, while their vulnerability to PGDM1400 was modestly reduced, suggesting its epitopes might partially overlap with those of CD4bs antibodies. These data show that observed resistance was not due to a generalized resistance mechanism or any non-specific growth advantages, and indicate that escape variants can readily be detected from a soft-randomized library swarm.

### HIV-1 can escape from CD4bs bNAbs through a wide range of diverse pathways

**Fig 5:**
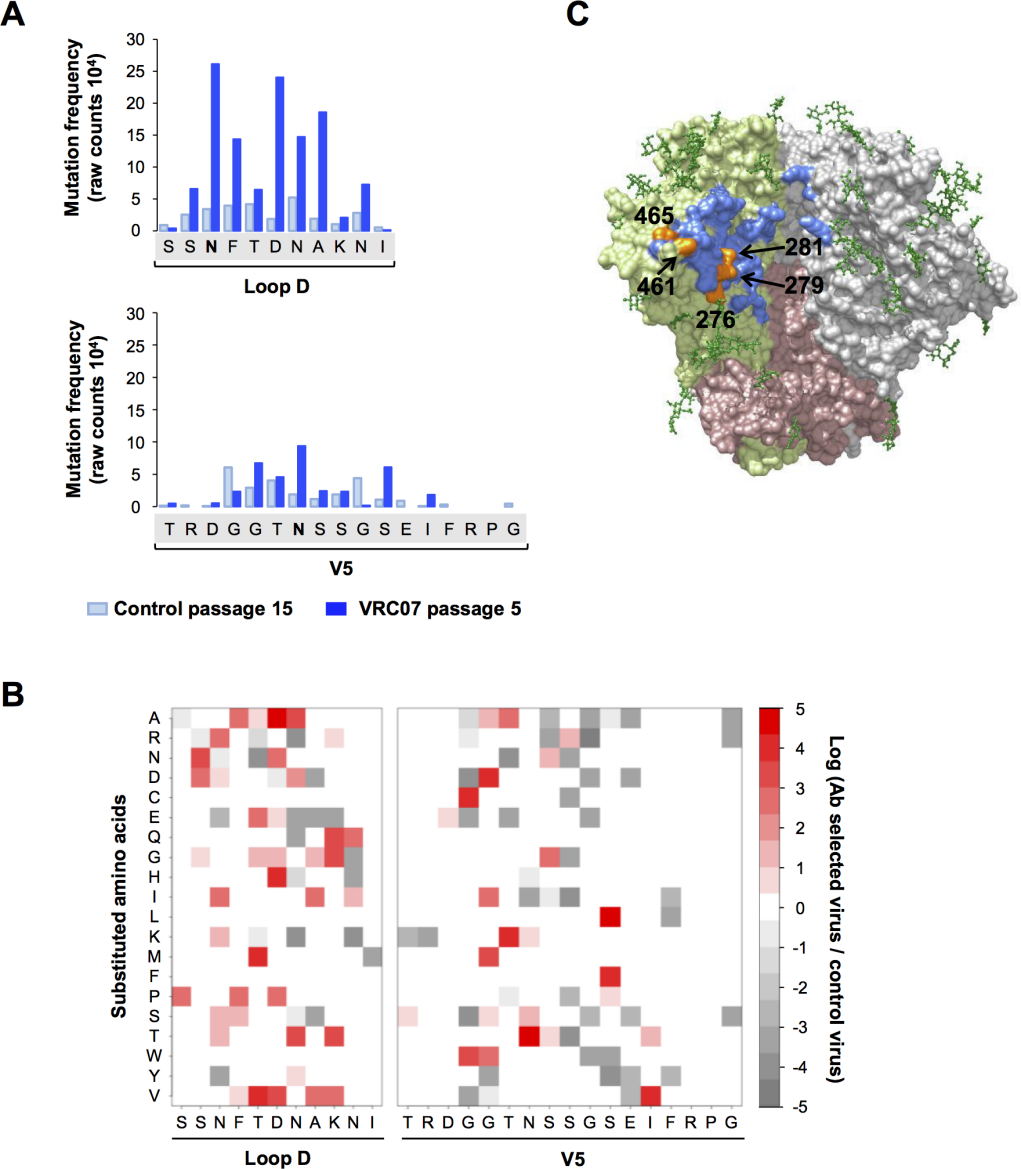
Alteration of Loop D residues is preferred to that of V5 residues for viral escape of CD4bs bNAbs. (**A**) The frequency of mutation at each amino acid position in the Loop D and V5 regions of the VRC07 selected passage 5 virus swarm. The top 150 sequences determined by their copy numbers detected through deep sequencing, was analyzed. Soft-randomized residues are shown on X-axis, and bold “N” indicates a putative N-glycosylation site. ‘Control passage 15’ virus is the library virus passaged in the absence of an antibody but the same number of times as the VRC07-selected viruses. The residue alterations in the control virus likely represent those advantageous for replication in GHOST-R3/X4/R5 cells. Mutations in Loop D appear to be more frequently selected than those in V5, and loss of glycosylation is one of the most frequently selected changes in both regions. (**B**) Amino acid substitutions in the Loop D and V5 regions of VRC07 selected passage 5 viruses are shown in 2-dimensional heat maps. The relative frequency of the substitutions of a residue is represented by a color gradient. (**C**) Three Loop D (N276, D279 and A281) and two V5 (N461 and S465) residues found most frequently substituted in escape variants are indicated in orange in a space-filling model of an Env trimer (PDB 5V8M). In this structure, Env is derived from a clade A isolate, BG505, and its residue 279 is N, and both 461 and 465 are T. Residues involved in the association of VRC07 are derived from the VRC07-bound Env monomer structure (PDB 4OLU) and are indicated in blue. One monomer of gp120 is shown in light green, 2^nd^ gp120 monomer in grey, and gp41 molecules in pink. N-glycans are represented as balls and sticks in dark green.

To identify the sequence variation that contributes to escape phenotype, VRC07 passage 5 swarm was then deep sequenced in the Loop D and V5 regions and compared to the sequences of the parental library swarm or that passaged 15 times in GHOST cells in the absence of any antibody (control passage 15). The total number of variants containing a substitution at the indicated soft-randomized residue are shown in Fig 5A, and their specific substitutions in Fig 5B. Although the original library swarm had fewer mutations in Loop D than in V5 region (Fig 2), higher number of Loop D mutations was detected in the escape variants (Fig 5A). Some substitutions are enriched in the control swarm, as expected, likely because they provided growth advantages in this experimental setting. The residues found most frequently substituted in the escape virus in this study are N276, D279 and A281 in the Loop D, and N461 and S465 in the V5 regions, similar to previous observations [7, 11, 25, 27, 40]. These residues are indicated in the crystal structure of an Env trimer (Fig 5C).

**Fig 6:**
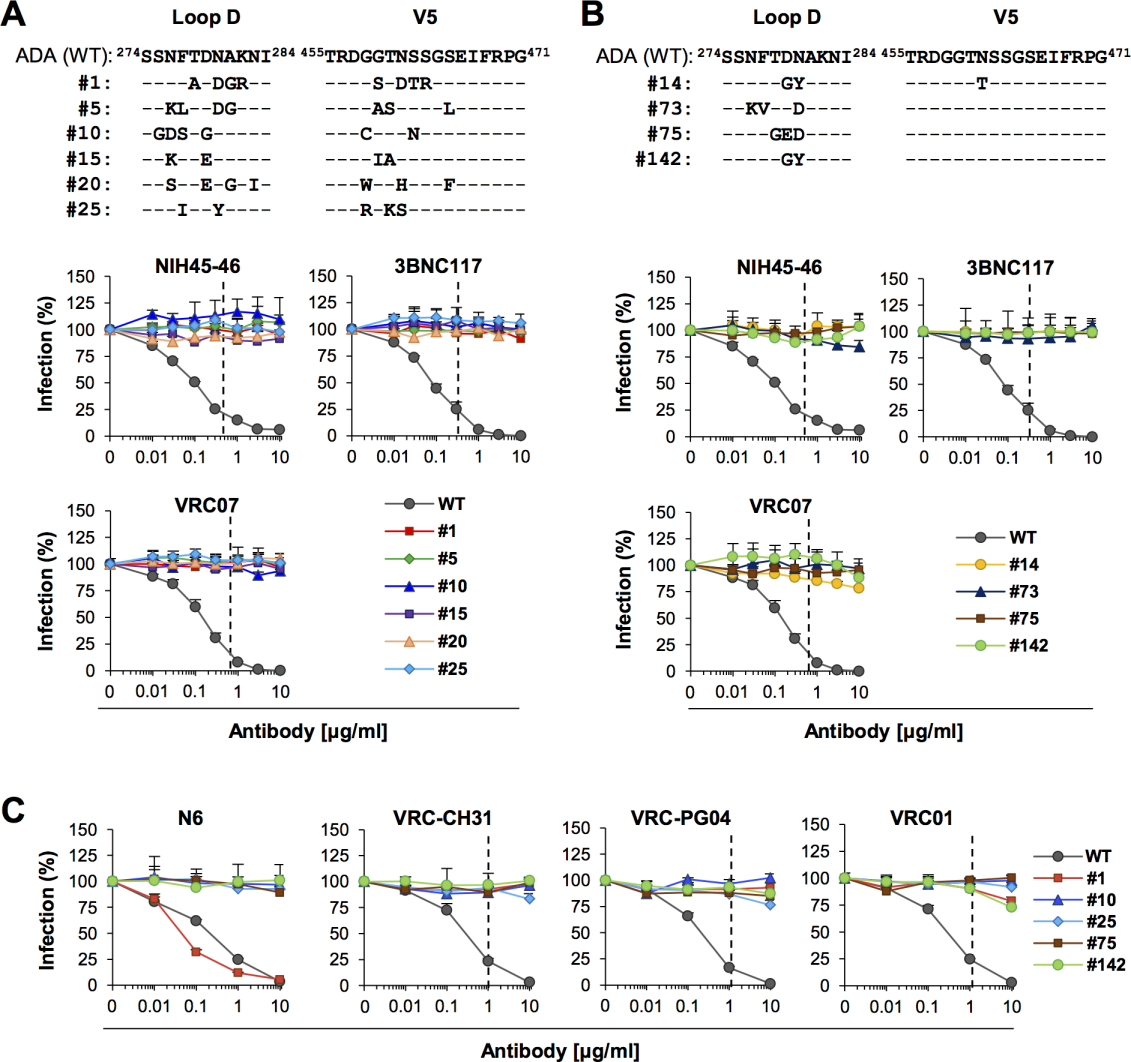
HIV-1 can escape from CD4bs bNAbs through a wide range of diverse pathways. (**A**) Upper panel: Loop D and V5 sequences of the six clones randomly chosen from top 25 of the VRC07 passage 5 swarm. Sequence ranking is based on copy numbers in the VRC07 passage 5 swarm, normalized by the copy number of individual sequence in the control swarm passaged the same number of times but without antibody (Control passage 15 swarm). Lower panel: Neutralization assays of these virus clones against the indicated antibodies. WT is the parental NL-ADA virus. (**B**) The sequences of Loop D and V5 (upper panel) and neutralization assays (lower panel) of four additional clones chosen from top 150 for the small number of mutations (2 or 3) they contain. (**C**) Neutralization assays of clonal viruses against additional CD4bs bNAbs N6, VRC-CH31, VRC-PG04 and VRC01. The dotted lines in (A)—(C) represent average IC_80_ values of antibodies for the ADA isolate. Averages ± SD of three independent experiments performed in duplicates are shown. Statistical significance compared with WT NL-ADA was calculated by two-way ANOVA and *p* values were <0.001 for all in (A)—(C).

**Fig 7:**
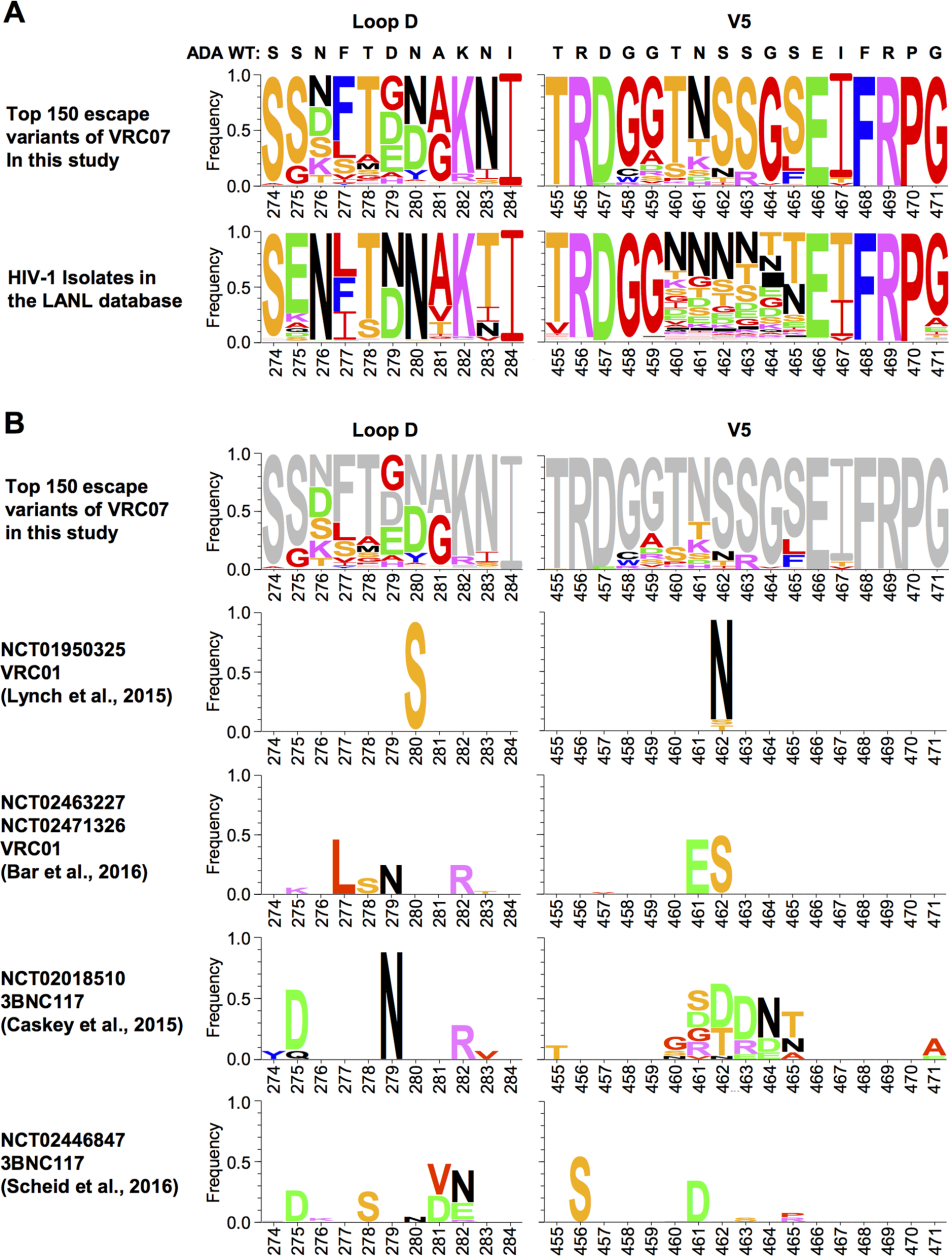
A subset of escape mutations selected from soft-randomized library are found in natural isolates and previously-identified *in vivo* escape variants. (**A**) Sequence analysis of the Loop D and V5 regions of our top 150 escape variants (top panel) and 5471 naturally-occurring HIV-1 isolates (bottom panel) was performed using the AnalizeAlign tool available at LANL (www.hiv.lanl.gov), and presented in logo plots. (**B**) Similar sequence analyses of these 150 escape variants (top panel) and the escape mutations detected in the clinical trials evaluating VRC01 or 3BNC117 is shown. Only four trials [6, 7, 11, 12], from which complete sequence information is available, are included in the analyses. For clarity, parental sequences and insertion mutations have been excluded. Clinical trial IDs, antibodies, and references are indicated for each analysis. Amino-acid numbers are based on HXB2 numbering.

To rank enriched sequences, copy number of each sequence found in VRC07 passage 5 swarm was normalized by the copy number of the same sequence detected in the control passage 15 swarm. To include in normalization the sequences that were detected in VRC07 passage 5 swarm but not in the control passage 15 swarm, a control copy number of 1.0 was added to all sequences. The sequences of the top 150 escape variants after normalization are listed in S3A Fig. The same sequences are presented in S3B Fig, with each residue marked with a different color to highlight their similarities and substitution patterns. To confirm the resistance of the escape variants to CD4bs antibodies, we first chose six clones (1, 5, 10, 15, 20 and 25) from the top 25. The sequences of these clones—771 bp fragments containing only the mutations found in the Loop D and V5—were synthesized and cloned first into the pBR322*-env* (ADA) using engineered NcoI and MluI sites (S1 Fig) and then into the pNL-ADA plasmid. Replication-competent clonal viruses were produced from 293T cells by transfecting corresponding plasmids. When assessed with the three antibodies they were selected against, we observed that all these virus clones were completely resistant to them (Fig 6A, lower panel). We also characterized several additional clones from the top 150 (S3 Fig), which contained a small number (2 or 3) of substitutions (Fig 6B, top panel). As shown in the lower panel of Fig 6B, most of these clones were also resistant to all three antibodies they were selected against. These results suggest that many, if not most, of the top 150 escape variants are resistant to all three antibodies. These clones were then tested against additional VRC01-class antibodies: N6, VRC-CH31, VRC-PG04 and VRC01. All clones except clone 1 were resistant to all of these antibodies (Fig 6C). Clone 1 was sensitive to N6 but resistant to all other antibodies. Of note, clone 142, bearing only two substitutions (D279G, N280Y), was resistant to all VRC01-class antibodies tested, including N6. Although D279G was not sufficient by itself to confer resistance to VRC07 (S4A Fig), more than half of known HIV-1 isolates contain an amino acid other than D at the residue 279 (Fig 7A and LANL sequence database), indicating high flexibility at this position.

Whereas none of the top 150 clones gained an N-linked glycan, a majority (136) has lost the well-characterized N-linked glycan at N276 in Loop D through a substitution either at N276 and/or at T278, and 66 clones have substitution either at N461 and/or at S463 in V5 region. The loss of a N276 glycan is consistent with the reports that this glycan is required for virus neutralization by VRC01-class antibodies [22, 29, 41]. While it was previously detected in *in vivo* escape variants [25, 27], most natural isolates maintain this glycan (Fig 7A and LANL sequence database). When examined, however, loss of the N-glycan at residue 276 alone did not confer resistance to VRC07 (S4B Fig). This mutation was also not necessary for resistance to other VRC01-class antibodies; indeed, three clones (25, 14 and 142) we tested retained the N-glycan at N276, but they were nonetheless resistant to all VRC01-class antibodies (Fig 6).

In addition to the aforementioned mutations at the residues 276, 279 and 461, several other mutations identified in our top 150 escape variants are found in naturally-occurring isolates (LANL sequence database), including L277, S278, I283, S460, T/S/K at reside 461, and N/T at 462 (Fig 7A). Although the sequence variation among different parental swarms in different patients complicates analysis, a subset of the mutations found in our escape variants has also been previously reported in the clinical trials evaluating CD4bs bNAbs, VRC01 and 3BNC117 [6, 7, 11, 12]. These include K276, L277, S278, R282, S460, S/D at residue 461, N/T at residue 462, and R463 (Fig 7B). Thus, some of the mutations identified through our approach are found in infected humans. Collectively, our data demonstrate that there are abundant and diverse pathways through which HIV-1 can escape CD4bs antibodies.

### Escape variants exhibit moderately slower growth than WT virus

**Fig 8:**
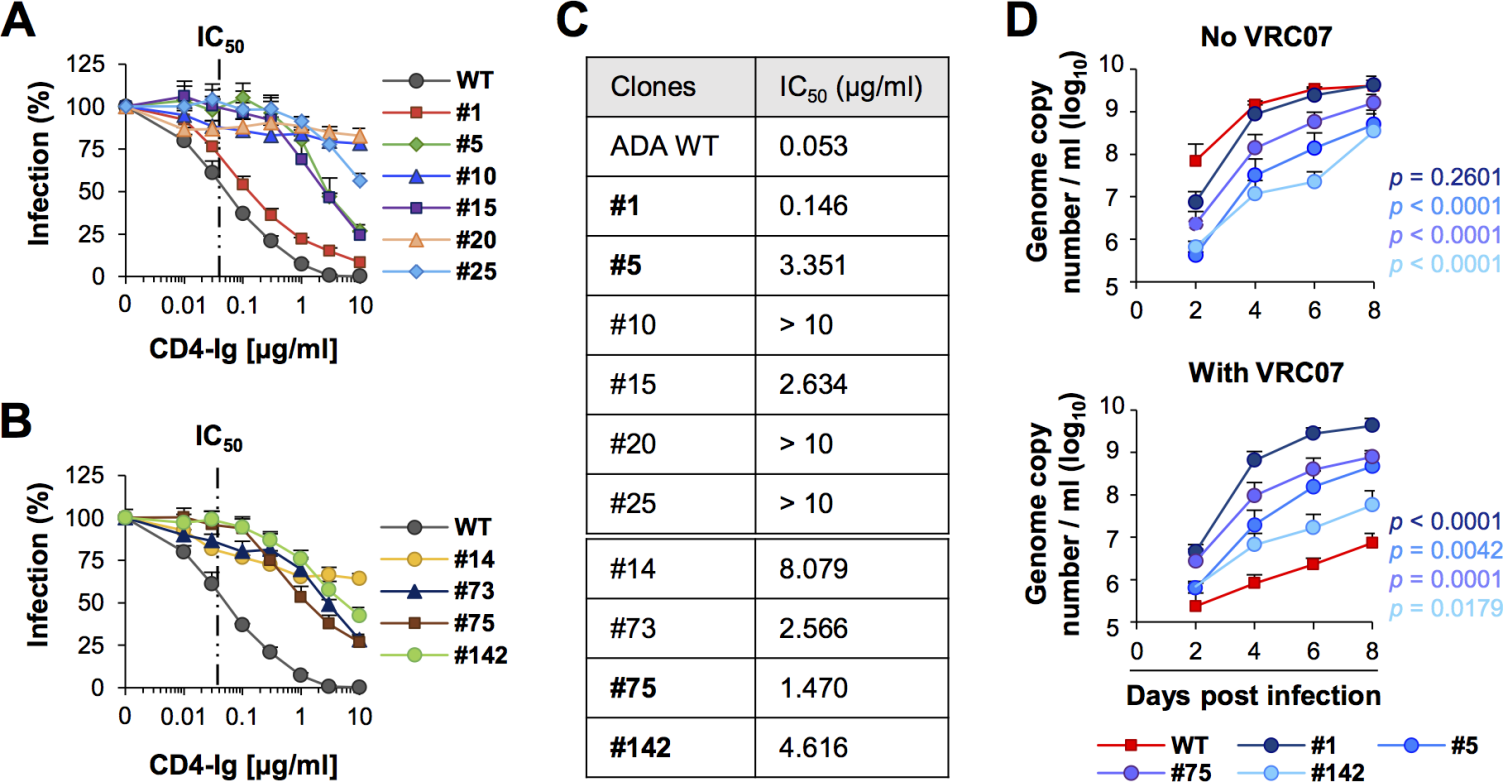
Escape variants exhibit slower growth than WT virus but outgrow WT virus in the presence of bNAbs. CD4-Ig neutralization assays of the virus clones 1, 5, 10, 15, 20 and 25 (**A**) or clones 14, 73, 75 and 142 (**B**) performed in TZM-bl cells. WT is the parental virus, NL-ADA. Averages ± SD of three independent experiments performed in duplicates are shown for (A) and (B). (**C**) IC50 values of CD4-Ig calculated from the inhibition assays shown in (A) and (B) for WT virus and each of indicated virus clones. The clones indicated by bold letters were characterized for their replication efficiency in the experiments shown in (D). (**D**) Replication efficiency of WT and indicated virus clones in CD4+ T cells in the presence and absence of 10 μg/ml VRC07. Progeny viruses were harvested every 2 days up until 8 days post infection and quantified by RT-qPCR. Averages ± SD of three independent experiments performed in duplicates are shown. Statistical significance was calculated using two-way ANOVA, compared WT to each clone and indicated with corresponding colors.

Many of CD4bs bNAb escape variants were shown to exhibit fitness cost [42, 43], because changes in the antibody epitopes are likely alter virus binding to CD4. Typically compensatory mutation emerge that restore fitness while maintaining resistance. To assess fitness, we first measured the ability of WT and escape variants to use CD4 by measuring their neutralization sensitivity to CD4-Ig (Fig 8A–8C). Infection of TZM-bl cells by WT virus and clone 1 was similarly inhibited by CD4-Ig. However, CD4-Ig less potently neutralized other escape clones, indicating that they have reduced affinity for cellular CD4. Next, we assessed their fitness in the CD4^+^ T cells prepared by activating peripheral blood mononuclear cells with phytohemagglutinin-L (Fig 8D). Progeny virus production was measured by RT-qPCR in the culture supernatants of the infected cells every two days until day 8 post infection. The growth difference between WT and escape clones was substantial, except clone 1, especially in early time points, indicating the fitness of most escape variants was compromised. However, progeny virus production by clone 1 reached similar level as that of WT virus by day 4. In addition, as was shown in Fig 6, whereas replication of escape variants was not affected by the presence of VRC07, more than 99% of WT replication was inhibited. These data are consistent with the observations repeatedly made in the clinical trials evaluating CD4bs bNAbs [6, 7, 10–12, 23] that although generally less fit than WT virus, once selected, the escape variants outcompete WT virus in the presence of bNAbs.

## Discussion

Most bNAbs are able to neutralize majority of known HIV-1 isolates *in vitro* and prevent a new infection or control an established infection in animal studies. It is well established, however, that resistant viruses easily emerge *in vitro* and *in vivo* in the presence of these antibodies (reviewed in [1, 4]). While much effort is focused on using passively administered bNAbs and eliciting bNAbs through vaccination, less effort has been dedicated to understanding how viral escape will impact the utility of those approaches. Comprehensive insight into the ways HIV-1 can escape bNAbs, and methods by which this escape potential could be rapidly assessed, are critical to the use of bNAbs in humans. Without such insight, it is difficult to determine whether an antibody will be therapeutically useful, how it might be improved, whether it would work best in concert with other antibodies or antiviral drugs, or whether its epitope would be a useful target for a therapeutic or prophylactic vaccine. Most importantly, such information is necessary to determine whether the use of bNAbs in humans will easily promote emergence and spread of resistant variants.

Although there are a number of reports describing *in vivo* bNAb escape mutations, such studies have been limited to only a few antibodies, and by no means comprehensive. Whereas these *in vivo* escape variants emerge rapidly, within a few weeks after antibody infusion [6–8, 11, 13, 24–26], conventional *in vitro* escape studies typically take much longer. These studies are slow because viral reverse transcriptase introduces only approximately one nucleotide mutation per viral genome per replication cycle. Because a typical antibody epitope is encoded by 1–2% of the genome, it takes at least 50 replication cycles to introduce a single relevant mutation into a virus population. To accelerate this process, Dingens et al. recently adopted codon mutagenesis, a scanning mutagenesis-based approach [44]. This method generates a library of the *env* gene encoding every possible single amino-acid change in the Env ectodomain. Although comprehensive, this approach has several disadvantages relative to the soft-randomization approach we used here. First, the costs and labor of library generation are markedly greater; library generation with codon mutagenesis requires hundreds of primer pairs and corresponding numbers of PCR reactions, whereas soft randomization uses only a single pair of primers and one PCR reaction per target region. More critically, with codon mutagenesis, one cannot identify escape variants bearing more than one mutation. It therefore can only identify short pathways of escape unique to the specific Env under study. In contrast, as we show here, soft randomization can identify many escape variants with multiple changes, better reflecting the pathways available to a huge number of highly diverse viruses in circulation. In fact, most previously characterized bNAb escape variants identified in *vivo* bear multiple mutation [6–8, 11, 13, 24–26]. Nonetheless, our approach has one key disadvantage relative to codon mutagenesis, namely it requires structural knowledge of the epitopes of the bNAbs under investigation, and excludes escape pathways involving changes outside of those epitopes. In the case of HIV-1 Env and bNAbs, however, the amount of available structural and functional data largely compensates for this limitation. As both approaches and their limitations are complementary, in the future, they may be combined to provide maximum insight into viral escape.

Here we extended soft-randomization to HIV-1 and diversified the regions encoding a key antibody epitope, and showed that this approach could create a library of functional replication-competent HIV-1 proviruses with a controlled numbers of amino acid substitutions in each Env. Using well-characterized VRC01-class antibodies as an example, we further showed that a disturbingly high number of such substitutions facilitated viral escape from those antibodies. In addition, a subset of the escape mutations identified in this study was also detected in *in vivo* studies and in natural isolates. For example, among the five residues found most frequently substituted in this study (N276, D279, A281, N461 and N465), loss of glycosylation at N276 was also observed in escape variants derived from humans and humanized mice [25, 27, 45]. This glycan is highly conserved (in 92% of known HIV-1 isolates, HIV Sequence Compendium 2017 [46]), and its loss further exposes the CD4-binding site to humoral immunity. However, loss of this glycan among escape variants is not unexpected because it participates in the binding of VRC01 antibodies [29, 41, 45]. On the other hand, glycosylation at N461 is present only in 27% of known isolates, indicating that mutations can be readily accommodated at this position. Over 60% of HIV-1 isolates listed in the HIV Sequence Compendium 2017 [46] has residues other than D at the position 279, indicating that variations at this position would also not be difficult, as indicated by its rapid evolution in an infected individual [27]. The similarities between the escape variants from our study and those derived from humans or humanized mice validate the utility of a soft-randomized library approach for rapidly assessing viral escape from a bNAb.

We observed a very high number of escape pathways from VRC01-class antibodies *in vitro*. This large number of escape pathways is important and problematic for the use of these antibodies in humans, for several reasons. First, it suggests that any effort to “checkmate” the virus by using a cocktail of antibodies, in which viruses escaped from one antibody are designed neutralized by others of the same class, is unlikely to be successful. There are simply too many possible escape pathways to cover them all. Second, our data make clear that the current panels of Env used to assess antibody breadth and potency do not in any way encompass the possible ways through which HIV-1 responds to bNAbs. Occasionally breadth is discussed as a surrogate for difficulty of escape, but our data indicate that these concepts are dissociable. For example, N6 is among the broadest CD4bs bNAbs thus far described, but the virus appears to have many available pathways to escape it (Figs 4B and 6C). In fact, in some cases an antibody may be broad because it is rare, and thus its epitope has not been under pressure to diversify. Third, our data indicate that there is considerable overlap in the ways viruses can become resistant to different VRC01-class antibodies. For example, resistance to N6 readily emerged when viruses were selected against other VRC01-class members. Thus population-level escape from VRC01 may easily promote escape from 3BNC117 or N6. Finally, because many different sets of substitutions can resist all VRC01-class antibodies, engineered vaccines designed to mainly elicit VRC01-class antibodies [47, 48] may not sufficiently suppress population-level escape, and therefore may need to be supplemented by constructs designed to elicit bNAbs targeting complementary epitopes [49, 50].

Although, as a proof of principle, we focused here only on VRC01-class antibodies and ADA isolate, HIV-1 likely has a similarly wide range of pathways of escaping other bNAbs. Indeed, escape might pose greater challenges with other bNAbs such as V2-loop/apex antibodies and 332-glycan antibodies, whose epitopes are less conserved and less functionally important than the CD4 binding site. One possible exception may be the antibodies recognizing the highly-conserved gp41 MPER epitope. A similar study with an MPER-region library may reveal greater constraints on escape than observed here for VRC01-class antibodies.

In summary, soft-randomization of the key epitopes of HIV-1 Env is a useful approach for rapidly and extensively identifying antibody-resistant viruses, and thereby provides important insights into the propensity of a bNAb to promote viral escape and the potential pathways of escape. Its first application to the CD4bs bNAbs in this study extends previous observations and shows that there are a large number of discrete pathways by which HIV-1 can escape all VRC01-class antibodies.

## Materials and methods

### Cells and plasmids

Human embryonic kidney (HEK)-293T cells were obtained from the American Type Culture Collection (ATCC, CRL-3216) and used to generate library and clonal viruses by transfection. The TZM-bl cells were obtained from NIH AIDS Reagent Program and used as an indicator cell line to measure the infectivity of various viruses [51]. Both cell lines were maintained in high-glucose Dulbecco’s minimal essential medium (DMEM) containing 10% fetal bovine serum (FBS). GHOST cells are derived from the human osteosarcoma cells line, HOS. GHOST (3) CCR3+CXCR4+CCR5+ cells were obtained from NIH AIDS Reagent Program and used to passage virus library in the presence of antibodies, and were maintained in DMEM containing 10% FBS, 500 μg/ml G418, 100 μg/ml hygromycin, and 1 μg/ml puromycin [52]. In later text, this media is referred as GHOST-cell complete media, and GHOST (3) CCR3+CXCR4+CCR5+ cells are referred as GHOST-R3/X4/R5. All cells were grown at 37°C under 5% CO2.

The gene for the envelope glycoprotein (*Env*) of HIV-1 ADA (GenBank AY426119.1) was cloned into pBR322 using SalI and BamHI sites, and used as a template to generate soft-randomized libraries. An AscI site was engineered between Loop D and V5 regions at amino acid positions 309–311 in order to combine independently generated Loop D and V5 libraries. The Env library from pBR322 was cloned into pNL4-3 proviral plasmid containing the *env* gene from ADA isolate (pNL-ADA), using SalI and BamHI sites, to generate a replication-competent virus library. Loop D and V5 fragments containing the escape mutations identified by deep sequencing were synthesized by Integrated DNA Technologies (IDT) and cloned into pBR322, and then into pNL-ADA. Plasmids encoding antibodies and CD4-Ig are described in Antibodies section.

### Antibodies

The broadly-neutralizing antibodies (bNAbs) used in this study are VRC01, 3BNC117, NIH45-46, VRC07, VRC-PG04, VRC-PG20, VRC-CH31, b12, N6, 10E8, 10–1074, PGDM1400. Of these, VRC01, 3BNC117, NIH45-46, VRC07, VRC-PG04, VRC-PG20, VRC-CH31, b12, N6 are CD4 binding-site (CD4bs) antibodies, 10E8 binds to the gp41 MPER epitope, 10–1074 binds a glycan on the V3 loop, and PGDM1400 recognizes an Env oligomer. IC_80_ and IC_50_ of these antibodies for ADA were obtained from the LANL database CATNAP (http://hiv.lanl.gov/catnap) [53], and are provided in S2B Fig. The expressor plasmids for antibodies NIH45-46, 3BNC117 and 10–1074 were kindly provided by Michael Nussenzweig (The Rockfeller University). Those for VRC01 and 10E8 were obtained from AIDS Reagent Program, and the rest were constructed by cloning the genes for the heavy- and light-chain variable regions synthesized by IDT into the plasmids encoding the constant regions of the light chain and human heavy chain of IgG1, as previously described [54]. GenBank numbers for the synthesized heavy and light chains are: VRC07 (H, KT365998.1; L, KM408147.1); VR-PG04 (H, JN159464.1; L, JN159466.1); VRC-PG20 (H, KF515514.1; L, KF515513.1); VRC-CH31 (H, JN159435.1; L, JN159438.1); N6 (H, KX595108; L, KX595112); b12 (H, AAB26315.1; L, AAB26306.1); PGDM1400 (H, KP006370.1; L, KP006383.1). All antibodies were produced in Expi293 Expression Medium (Life Technologies) by transfecting Expi293 cells with the corresponding expressor plasmids. Cells were grown for three days at 37°C with humidified air containing 8% CO2 on an orbital shaker platform rotating at 125 rpm. Proteins were purified from the culture supernatants using Protein A-Sepharose beads.

### Generation of soft randomized library

The protocol for soft randomization was previously described [36]. In brief, primers were designed for Loop D (amino acids 274–284 by HXB2 numbering) and V5 (amino acids 455–471) regions of ADA *env* gene. The soft-randomizing primer for Loop D was synthesized by hand-mixing 88% of the original nucleotide and 4% each of the other three nucleotides (88:4:4:4) for the first two positions of each codon of the 11 residues. A ratio of 91:3:3:3 was used for the longer (17 residues) V5 region. For the wobble positions of the Loop D primer, an equimolar mix (50:50) of G and T was used for amino acids encoded by 4 or 6 codons. For the wobble positions of other codons, the ratios of 88:4:4:4 (for Loop D) or 91:3:3:3 (for V5) was used. Both the 5’ and 3’ ends of the primers were extended outside the soft-randomized regions to match the melting temperature of pairing primers. Soft-randomizing primers were phosphorylated at the 5’ ends. The sequences of the soft-randomizing primers and pairing primers are shown in Fig 1B.

The Loop D and V5 soft randomization was performed independently via whole-plasmid PCR of pBR322 carrying SalI-BamHI fragment of ADA *env* gene, using Q5 HotStart DNA Polymerase (New England Biolabs). 1 μg of DpnI-digested and purified PCR product was ligated in 40 μl with 0.1 μl of concentrated T4 ligase (New England Biolabs) at 16°C overnight. 1 μg of ethanol-precipitated ligate was electroporated at 1700V into 25 μl of Electrocompetent NEB 10-beta (New England Biolabs), and the culture was grown in S.O.C. media with shaking at room temperature for 1.5 hours. The culture was then added to 300 mL Lennox broth containing 50 μg /ml ampicillin and grown with shaking until the culture reached an OD_600_ of 1.7. Chloramphenicol was added to final 170 μg/mL and the culture was grown for additional 36 h. To combine Loop D and V5 libraries, V5 library was subcloned into pBR322-Env (ADA) containing Loop D library, using AscI, engineered between Loop D and V5 regions, and BamHI sites. Ligation, DNA precipitation, electroporation, and plasmid preparation were performed as described above. pNL-ADA library was then constructed by subcloning the SalI-BamHI fragment of pBR322-Env(ADA) containing soft-randomized Loop D and V5 regions. Ligation, DNA precipitation, electroporation and plasmid preparation were again performed as described above. A total of 24 x 300 ml cultures, each 300 ml of which was derived from 1 ug ligate electroporated into 25 μl of NEB 10-beta, were grown to an OD_600_ 0.8–1.0. DNA was prepared as described above, pooled and used as the final pNL-ADA-Lib.

### Virus production

WT pNL-ADA and its library, and all clonal viruses (sequences are shown in S3 Fig) were produced by calcium phosphate transfection of HEK-293T cells with corresponding plasmids, and cells were grown in DMEM containing 10% FBS. The virus-containing supernatants were harvested 48 hours post infection (hpi), aliquoted, and stored at -80°C. All experiments involving replication-competent viruses were performed in biosafety level 3 laboratory following the protocols approved by the Institutional Biosafety Committee of The Scripps Research Institute.

### Selection of antibody-resistant viruses

Library swarms, produced by transient transfection of HEK-293T described above, or obtained from a previous passage were incubated in GHOST-cell complete media for 30 minutes with indicated concentrations of NIH45-46, 3BNC117 or VRC07 antibody. Antibody concentrations used for selection are provided in S2A Fig for each antibody and passage. For this study, we chose virus dilution that yielded 60–70% infection of GHOST cells at 48–72 hpi. The virus-antibody mixture was then added to GHOST-R3/X4/R5 cells pre-seeded at ~40% confluence in 10 x T75 flasks. After 6–8 h, cells were washed twice with Phosphate-Buffered Saline (PBS) and further incubated in GHOST-cell complete media containing the same concentration of an antibody used for selection. The Tat-regulated GFP expression in GHOST cells was used to assess virus infection levels. When infection level reaches 60–70%, the virus-containing culture supernatants were harvested, spun to remove cell debris, aliquoted, stored in the -80°C, and used for subsequent viral passages or RNA extraction for deep sequencing.

### Next generation sequencing and analysis

RNA was extracted from 250 ul of cell-free culture supernatants of library or antibody-escaped swarms, using RNAqueous Total RNA Isolation kit (Ambion, ThermoFisher Scientific). cDNA was synthesized using High Capacity cDNA Reverse Transcription Kit (Applied Biosystems) using a gene-specific primer (5’ GAACCCAAGGAACATAGCTCCTATC 3’). An *env* fragment encompassing Loop D and V5 regions was amplified via 18 cycles of PCR reaction using Takara Taq Hot-Start DNA polymerase (Takara Bio Inc.) and ADA-Env-F3 (5’ GGCAGTCTAGCAGAAGAAGAGGTAGTAATTAG 3’) and ADA-Env-B3 (5’ CACTTCTCCAATTGTCCCTCATATCTCCTC 3’) primers. The PCR products (amplicons) were purified by AMPure XP beads (Beckman Coulter) at a bead to sample ratio of 3:2, quantified using Qubit dsDNA HS (high sensitivity) assay kit (Life Technologies) and run on the DNA high sensitivity chip in the Bioanalyzer 2100 (Agilent) to confirm their length. 100ng of these amplicons were then end-repaired, 5’ phosphorylated, and A-tailed at the 3’ ends using TruSeq Nano kit (FC-121-4001, Illumina). Next, these amplicons were ligated with illumina barcoded-partial adapters, size selected using AMPure XP beads, and further amplified for 4 PCR cycles. The final products were validated on the Bioanalyzer 2100 and quantified using Qubit dsDNA HS assay, pooled at equimolar and sequenced by paired-end 150bp reads on the Illumina MiSeq at the Scripps Genomics Core Facility in La Jolla, California.

The reads were trimmed of adapters, and cropped to isolate Loop D and V5 fragments. These sequences were translated, filtered for open-reading frames by eliminating those with stop codons and unidentifiable amino acids, and analyzed using in-house python scripts. To avoid removal of rare but intended mutations, reads were not corrected for the low level of errors that are potentially introduced during reverse transcription, PCR and sequencing. Enrichment was determined by normalizing the copy number of each sequence found in VRC07 passage 5 swarm by the copy number of the same sequence detected in the control passage 15 swarm. To include the sequences in normalization, which were detected in VRC07 passage 5 swarm but not in the control swarm, a control copy number of 1 is added to all sequences.

### Neutralization assay

Neutralization assays were performed in TZM-bl cells, as described previously [51]. To use TZM-bl cells, antibiotics were removed from virus stocks by growing them in GHOST cells in the absence of antibiotics. This process also removed antibodies present in the culture supernatants. Virus-containing supernatants were harvested at 48–72 hpi, spun to remove debris, aliquoted, and used for neutralization assays. Viruses were incubated with 0–10 ug/ml of antibodies at room temperature for 30 minutes and then added to TZM-bl cells plated at 10,000 cells per 96 well one day before the assay. At 48 hpi, infection levels were measured by luciferase assays using Luc-Pair Firefly Luciferase HS assay kit (GeneCopoeia). The relative light unit was read at 575 nm using a Victor X3 plate reader (PerkinElmer).

### Virus growth in CD4^+^ T cells

To assess virus fitness, the growth of WT virus or virus clones that are resistant to bNAbs was assessed in CD4^+^ T cells. Cryopreserved human peripheral blood mononuclear cells (PBMC, StemCell Technologies), were thawed and incubated in RPMI containing 15% FBS and 20 U/ml human IL-2 (Roche) for overnight. Next day, cells were enriched with CD4^+^ T cells population by negative selection using Human CD4^+^ T Cell Isolation kit (Biolegend) and activated with 1 μg/ml PHA-L (Sigma) for 48 hours in RPMI supplemented with 15% FBS, 20 U/ml IL-2 at 1 x 10^6^ cells/ml. Viruses (5 x 10^8^ genome copy number, quantified by RT-qPCR) were pre-incubated for 20 min at room temperature with or without 10 μg/ml VRC07 in 100 μl RPMI supplemented with 15% FBS and 20 U/ml IL-2, and added to 1.5 x 10^5^ PHA-L-activated CD4^+^ T cells in 150 μl. After 6 h incubation at 37°C, cells were washed with PBS and resuspended in 500 μl fresh RPMI containing 15% FBS with or without 10 μg/ml VRC07. Every 2 days, 180 μl of supernatant was harvested and replaced with same amount of fresh media. RNA was extracted from 150 μl these supernatants using TRIzol LS (Ambion), and cDNA synthesized using High Capacity cDNA Reverse Transcription kit (Applied Biosystems) and an ADA *Env*-specific primer (5’-GAACCCAAGGAACATAGCTCCTATC-3’). Probe qPCR was performed using iTaq Universal Probes Super mix (Bio-Rad), ADA-*Env*-qPCR-sense (5’-CAAAGCCTAAAGCCATGTGTAAA-3’) and ADA-*Env*-qPCR-antisense (5’-CTCCTCTCATTCCCTCACTACTA-3’), primers, and ADA-*Env*-qPCR probe (5’-/56-FAM/CCCATCCTG/ZEN/TGTTACTTTAAATTGCACTGA/3IABkFQ/-3’) in CFX96 Touch Real-Time PCR Detection System (Bio-Rad).

### Comparative sequence analysis

The frequency of mutations in the Loop D and V5 regions is analyzed using the AnalizeAlign tool available at LANL (www.hiv.lanl.gov). The sequences included in the analyses are: 5471 natural isolates available at LANL database, 298 Loop D and 250 V5 sequences of *in vivo* escape variants previously identified in the clinical trials for CD4bs antibodies, VRC01 and 3BNC117 [6, 7, 11, 12], and the top 150 escape variants identified in this study. Because of the large number of parental sequences in *in vivo* studies, parental residues are excluded from the analyses of *in vivo* escape mutations. Insertion mutations found in some of *in vivo* variants are also excluded.

### Data and statistics

Statistical analysis of the data was performed using GraphPad Prism software. The difference between groups for all neutralizing assays and virus growth curves in CD4^+^ T cells were tested using a two-way ANOVA. The null hypothesis was rejected when *p*<0.05 in all cases.

## Supporting information

S1 Fig Construction of a pNL4-3 proviral library soft randomized in the Loop D and V5 regions. Loop D and V5 libraries are separately generated on pBR322 carrying the SalI-BamHI fragment of the *env* gene derived from ADA isolate. Two libraries were combined using engineered NcoI, AscI and MluI around Loop D and V5 regions. SalI-BamHI fragment of the combined library was then subcloned into the pNL4-3 proviral plasmid containing the *env* gene of ADA isolate (pNL-ADA).
(TIF)

S2 Fig Concentrations of antibodies used to derive escape viruses and IC_50_ and IC_80_ values for ADA isolate of the bNAbs used in this study. (**A**) The concentrations of antibodies used to derive escape variant viruses are listed for each virus and passage. Note that the IC_80_ value for VRC07 used in the selection study is slightly different from that presented in S2B Fig, due to an update in the LANL database CATNAP after this experiment were performed. (**B**) The IC_50_ and IC_80_ values for ADA isolate (http://hiv.lanl.gov/catnap). N.A.: Not available.
(TIF)

S3 Fig Loop D and V5 sequences of the top 150 clones of variant viruses escaped from CD4bs bNAbs. (**A**) The copy number of each sequence found in VRC07 passage 5 swarm was normalized by the copy number of the same sequence detected in the control swarm. Control swarm is the parental NL-ADA library passaged 15 times in total in the absence of any antibody. The sequences that were detected in VRC07 passage 5 swarm, but not in the control swarm, were assigned an arbitrary copy number of 1.0 for this normalization. To compensate this, 1.0 is added to all other sequences whose copy number in the control swarm is 1.0 or higher. (**B**) The same sequences as in (A) are shown with each amino acid presented in a different color to highlight their similarities and shared substitutions. Figure is generated using the Pixel tool available at LANL (www.hiv.lanl.gov).
(PDF)

S4 Fig A mutation at the residue N276 or D297 alone is not sufficient to confer resistance to VRC07. Neutralization assays against VRC07 of (A) D297G mutation, which is one of the two found in the clone 142, and (B) N276R glycosylation mutation. Averages ± SD of three independent experiments performed in duplicates are shown.
(TIF)
