# Supplementary figures and images for: Diverse pathways of escape from all well-characterized VRC01-class broadly neutralizing HIV-1 antibodies
Source: PLoS Pathog. 2018 Aug 20;14(8):e1007238. doi: 10.1371/journal.ppat.1007238 (PMC6117093; doi:10.1371/journal.ppat.1007238)

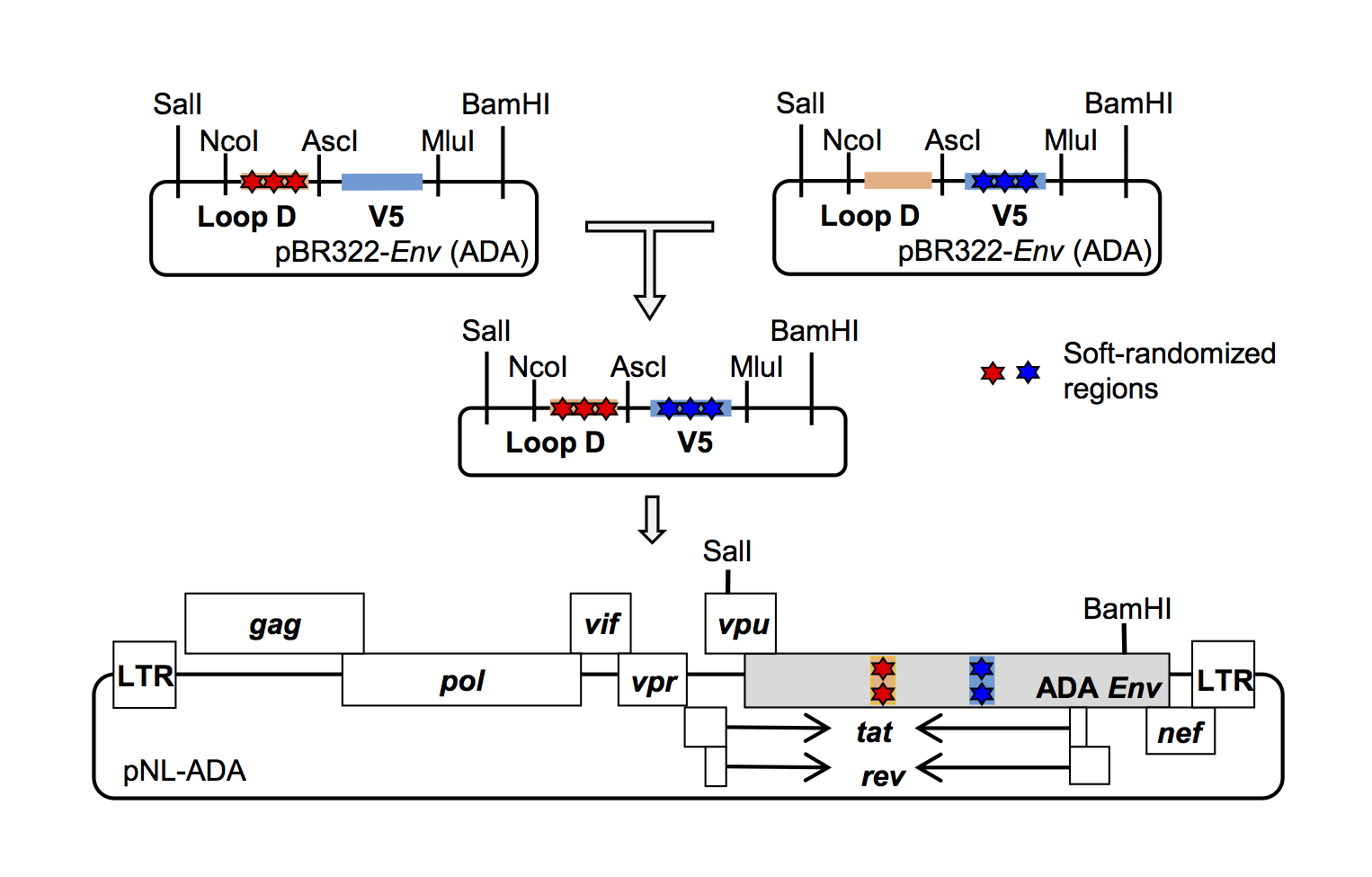

Supplement: S1 Fig — Loop D and V5 libraries are separately generated on pBR322 carrying the SalI-BamHI fragment of the env gene derived from ADA isolate. Two libraries were combined using engineered NcoI, AscI and MluI around Loop D and V5 regions. SalI-BamHI fragment of the combined library was then subcloned into the pNL4-3 proviral plasmid containing the env gene of ADA isolate (pNL-ADA). (TIF) [file ppat.1007238.s001.tif]

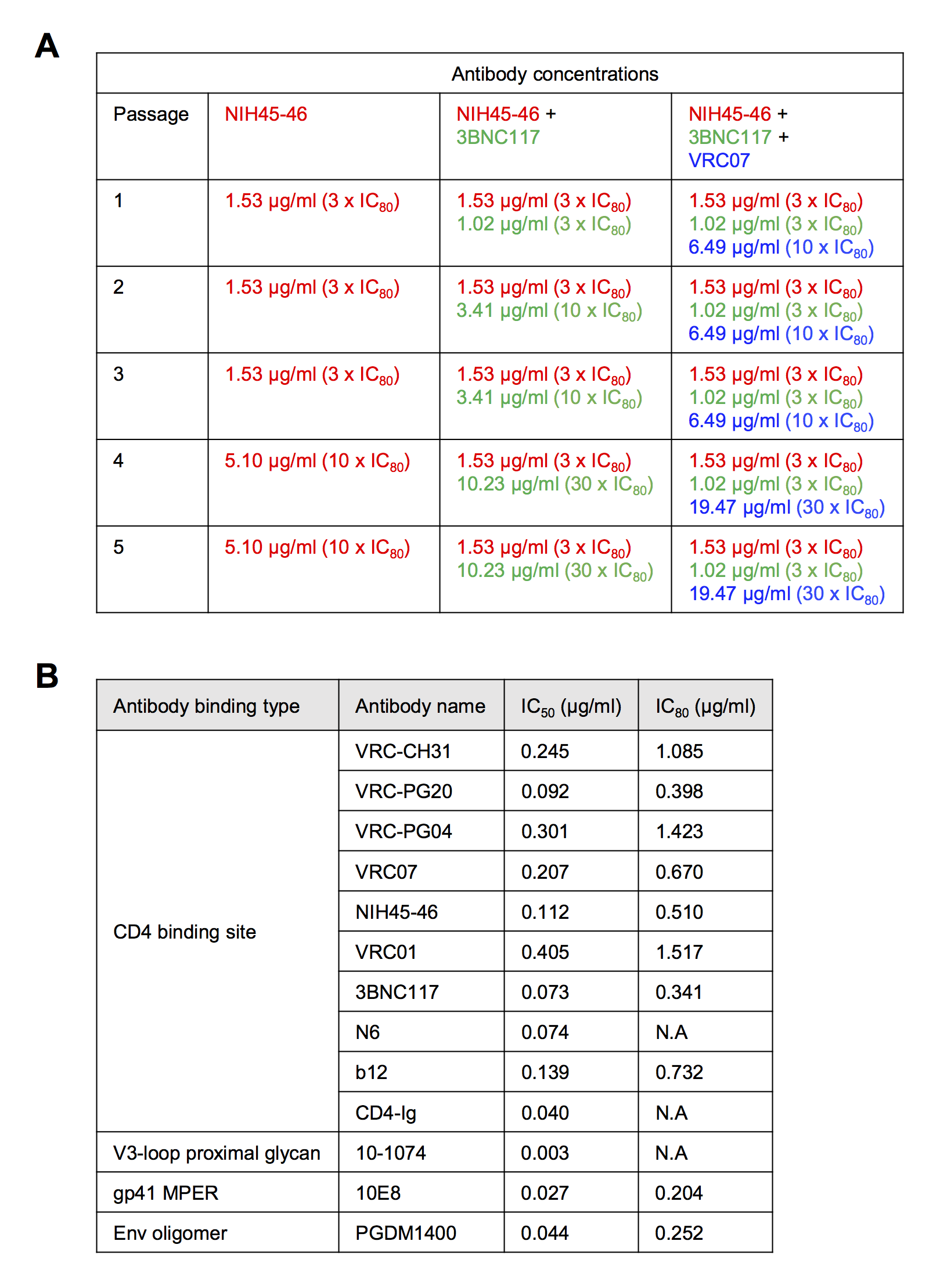

Supplement: S2 Fig — (A) The concentrations of antibodies used to derive escape variant viruses are listed for each virus and passage. Note that the IC80 value for VRC07 used in the selection study is slightly different from that presented in S2B Fig, due to an update in the LANL database CATNAP after this experiment were performed. (B) The IC50 and IC80 values for ADA isolate (http://hiv.lanl.gov/catnap). N.A.: Not available. (TIF) [file ppat.1007238.s002.tif]

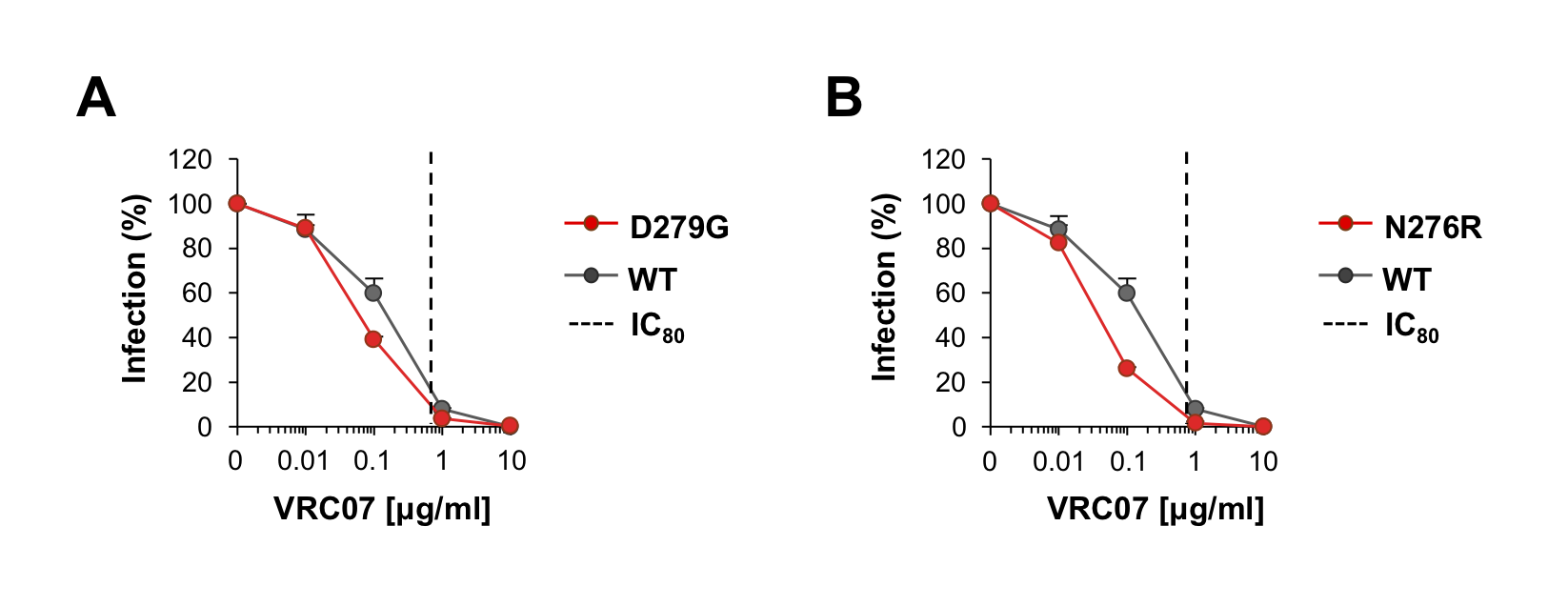

Supplement: S4 Fig — Neutralization assays against VRC07 of (A) D297G mutation, which is one of the two found in the clone 142, and (B) N276R glycosylation mutation. Averages ± SD of three independent experiments performed in duplicates are shown. (TIF) [file ppat.1007238.s004.tif]
