# Supplementary material for: Diverse pathways of escape from all well-characterized VRC01-class broadly neutralizing HIV-1 antibodies
Source: PLoS Pathog. 2018 Aug 20;14(8):e1007238. doi: 10.1371/journal.ppat.1007238 (PMC6117093; doi:10.1371/journal.ppat.1007238)
Supplement: S3 Fig — (A) The copy number of each sequence found in VRC07 passage 5 swarm was normalized by the copy number of the same sequence detected in the control swarm. Control swarm is the parental NL-ADA library passaged 15 times in total in the absence of any antibody. The sequences that were detected in VRC07 passage 5 swarm, but not in the control swarm, were assigned an arbitrary copy number of 1.0 for this normalization. To compensate this, 1.0 is added to all other sequences whose copy number in the control swarm is 1.0 or higher. (B) The same sequences as in (A) are shown with each amino acid presented in a different color to highlight their similarities and shared substitutions. Figure is generated using the Pixel tool available at LANL (www.hiv.lanl.gov). (PDF) [file ppat.1007238.s003.pdf]

A

	Loop D (274–284)	V5 (455–471)	% of total
ADA (WT):	SSNFTDNAKNI	TRDGGTNSSGSEIFRPG	–
#1	----A-DGR--	----S-DTR--------	1.07
#2	--T--A-G---	----AS----L------	0.28
#3	--T--A-G---	------T----------	0.27
#4	-GDS-G-----	----D-T----------	0.27
#5	--KL--DG---	----AS----L------	3.13
#6	----MG-G---	---W--H---F------	0.22
#7	----GED----	---C---N---------	0.60
#8	--D--ED----	----AS----L------	0.19
#9	-NGL--Y-R--	------T----------	0.19
#10	-GDS-G-----	---C---N---------	0.38
#11	--S--E-G-I-	----AS----L------	0.75
#12	--TY-GSGR--	------T----------	0.36
#13	--K---D----	---R-KS----------	0.17
#14	-----GY----	------T----------	0.52
#15	--K--E-----	----IA-----------	0.17
#16	---VVH-----	-----------------	0.84
#17	--TL-G-----	----R-K----------	0.17
#18	--S--E-G-I-	------K-R-F------	0.47
#19	----A-DGR--	----AS----L------	0.16
#20	--S--E-G-I-	---W--H---F------	0.16
#21	-GDS-G-----	------T---L------	0.62
#22	-GDS-G-----	----AS----L------	0.73
#23	--S--GAT---	-----------------	0.58
#24	A-S-P-D----	-----------------	0.29
#25	---I--Y----	---R-KS----------	0.72
#26	--S--E-G-I-	------S-----T----	1.95
#27	-GDS-G-----	----V--I---------	0.14
#28	-GDS-G-----	------T----------	2.03
#29	-GDAKNIIVQL	------T----------	0.13
#30	----M-DG---	------T----------	0.13
#31	--S--E-G-I-	---C---N---------	0.39
#32	-GDS-G-----	--E-D-------V----	0.13
#33	-----GY----	------K-R-F------	1.00
#34	-GDS-G-----	---W--H---F------	0.12
#35	--KL--DG---	------T----------	0.24
#36	----GED----	----AS----L------	0.12
#37	--S--E-G-I-	----S-DTR--------	0.23
#38	--SLSG-G---	S---------P------	0.77
#39	--S--E-G-I-	----V--I---------	0.11
#40	----GED----	------T----------	0.11
#41	-----GY----	----AS----L------	0.21
#42	--D--ED----	------T----------	0.73
#43	--KL--DG---	------K-R-F------	0.10
#44	-----GY----	---C---N---------	0.10
#45	----A-DGR--	------T----------	0.10
#46	--S--E-G-I-	--E-D-------V----	0.10
#47	----M-DG---	--E-D-------V----	0.71
#48	--T--H--Q--	-----P--R--------	0.10
#49	-GDS-G-----	----S-T----------	0.29
#50	--S--E-G-I-	------T----------	0.63
#51	--DS-G-G---	------T----------	0.35
#52	--SLSG-G---	-----------------	0.96
#53	--KL--DG---	------S-----T----	0.09
#54	--K--ET-GS-	-----------------	0.26
#55	--D---DG---	----R-K----------	0.26
#56	----EEDG---	-----------------	0.09
#57	-G----Y-T--	------T----------	0.08
#58	--KL--DG---	----S-DTR--------	0.08
#59	P-I-GG-V---	-----------------	0.08
#60	-RK---D--S-	-----------------	0.17
#61	----RP-----	-----------------	0.08
#62	-GDS-G-----	------S-----T----	0.16
#63	--S--E-G-I-	S---------P------	0.08
#64	----M-DG---	---C---N---------	0.08
#65	--DS-G-G---	----AS----L------	0.08
#66	----M-DG---	----AS----L------	0.16
#67	--KL--DG---	----R-K----------	0.08
#68	--D--ED----	------K-R-F------	0.07
#69	--KL--DG---	---C---N---------	0.14
#70	--DS-G-G---	----------V------	0.21
#71	----PG-G---	-----------------	0.42
#72	---VVH-----	----AS----L------	0.07
#73	--KV--D----	-----------------	2.46
#74	-GDS-G-----	----R-K----------	0.21
#75	----GED----	-----------------	0.90
#76	--T--A-G---	---C---N---------	0.14
#77	--S--E-G-I-	-----------------	5.90
#78	--D--ED----	---A---G--A------	0.14
#79	-----GY----	------S-----T----	0.07
#80	-GDS-G-----	-----P-N-V--T----	0.07
#81	--KL--DG---	---W--H---F------	0.07
#82	--SLSG-G---	----AS----L------	0.13
#83	--SLSG-G---	---C---N---------	0.07
#84	-GDS-G-----	----S-DTR--------	0.26
#85	-GDL-G-G-S-	-----------------	0.91
#86	--KV--D----	------K-R-F------	0.06
#87	----M-DG---	-----------------	0.70
#88	---I--Y----	------T----------	0.06
#89	--D--ED----	----S-DTR--------	0.06
#90	----M-DG---	------K-R-F------	0.06
#91	-GDLAH-----	----AS----L------	0.06
#92	----SAD-R--	---WAS-----------	0.12
#93	--D--ED----	------S-----T----	0.06
#94	--T--A-G---	---W--H---F------	0.06
#95	--SLSG-G---	------T----------	0.12
#96	-GDS-G-----	------K-R-F------	0.42
#97	--K---DG---	----AS----L------	0.12
#98	----A-DGR--	-----------------	0.53
#99	--KV--D----	------T----------	0.17
#100	-GDS-G-----	-----------------	3.06
#101	-GDS-G-----	----M------------	0.17
#102	--T--A-G---	-----------------	3.78
#103	--KV--D----	------S-----T----	0.06
#104	--KL--DG---	---R-KS----------	0.06
#105	--S--E-G-I-	----R-K----------	0.28
#106	--S----R-T-	---R-I-----------	0.05
#107	--SI--D----	---W-----D-------	0.06
#108	--S--GAT---	----AS----L------	0.05
#109	A-S-P-D----	----AS----L------	0.05
#110	--S--E-G-I-	----D------------	0.11
#111	--SI--D----	----M------------	0.16
#112	-----GY----	-----P--R--------	0.05
#113	-----GY----	--E-D-------V----	0.05
#114	--KV--D----	----AS----L------	0.21
#115	----AED----	----AS----L------	0.05
#116	--K---DG---	---C---N---------	0.05
#117	--D--ED----	---C---N---------	0.10
#118	----AED----	------T----------	0.05
#119	--S--H-----	----D------------	0.05
#120	--TY-GSGR--	------T---L------	0.05
#121	--D--ED----	-----P--R--------	0.05
#122	----GED----	------K-R-F------	0.10
#123	--T--A-G---	------K-R-F------	0.10
#124	A-R-KGH----	-----------------	0.10
#125	-GDLAH-----	------T----------	0.15
#126	----MG-G---	-----------------	0.10
#127	--SLSG-G---	------K-R-F------	0.05
#128	----M-DG---	----S-DTR--------	0.05
#129	-----A-G-T-	----V--I---------	0.05
#130	-GYY--D----	-----------------	0.05
#131	----AED----	----D-YN---------	0.05
#132	----A-DGR--	------K-R-F------	0.05
#133	-----EA-R--	---------------S-	0.05
#134	--D--ED----	-----------------	1.19
#135	--D--ED----	----R-K----------	0.05
#136	-GDLAH-----	-----------------	0.47
#137	--D--ED----	-----R-----------	0.05
#138	----A-DGR--	----R-K----------	0.05
#139	--DS-G-G---	-----------------	0.37
#140	--KL--DG---	-----------------	1.26
#141	--K--ET-GS-	----V--I---------	0.05
#142	-----GY----	-----------------	1.22
#143	-----GY----	---W--H---F------	0.04
#144	-GDS-G-----	-----P-T-V-------	0.04
#145	--SI--D----	-----------------	0.13
#146	--K---DG---	------T----------	0.09
#147	--S--K-G-I-	-----------------	0.04
#148	-GDL-G-G-S-	---C---N---------	0.04
#149	--T--H--Q--	-----------------	0.13
#150	-GDL-G-G-S-	----AS----L------	0.08

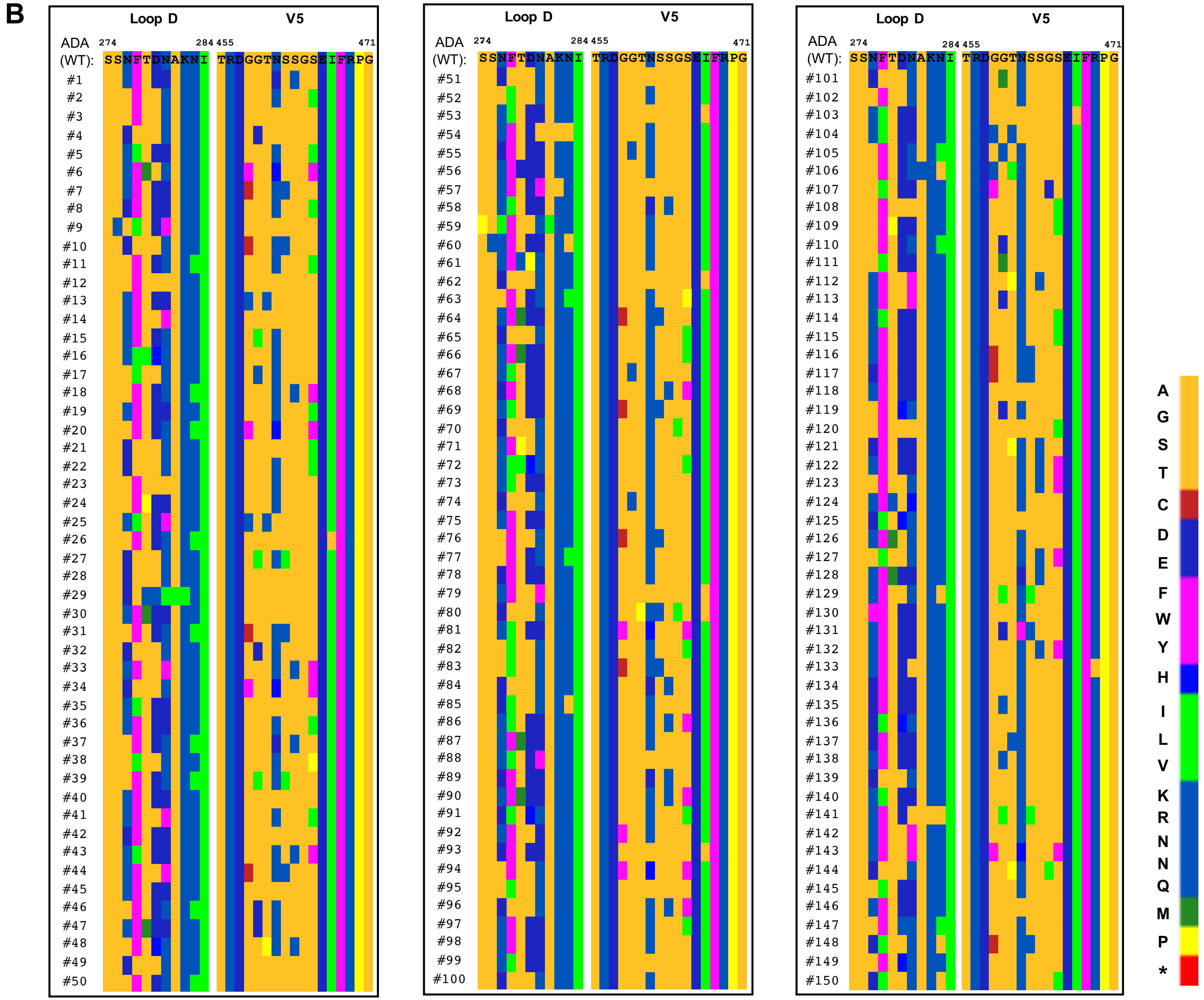

B
Loop D
V5
ADA (WT):
274
284
455
471
SSNFTDNAKNI
TRDGGTNSSGSEIFRPG
#1 – #150
A
G
S
T
C
D
E
F
W
Y
H
I
L
V
K
R
N
N
Q
M
P
*
